# Association between light at night and the risk of child death in sub-saharan Africa: a cross-sectional analysis based on DHS data

**DOI:** 10.1186/s12889-023-17284-1

**Published:** 2023-11-29

**Authors:** Xinyue Li, Jovine Bachwenkizi, Renjie Chen, Haidong Kan, Xia Meng

**Affiliations:** 1 School of Public Health, Key Laboratory of Public Health Safety of the Ministry of Education and Key Laboratory of Health Technology Assessment of the Ministry of Health, Fudan University, Shanghai, 200302 China; 2 Department of Environmental and Occupational Health, Muhimbili University of Health and Allied Sciences, Dar es Salaam, Tanzania

**Keywords:** Light at night, Under-five child mortality, Africa, Urbanization

## Abstract

**Background:**

The high under-five mortality rate (U5MR) in Africa is a significant public health concern. Previous studies have found that satellite retrieved light at night (LAN) data with long-term and global coverage can be used as a proxy for socio-economic development and urbanization. Currently, few studies on the effects of LAN on child mortality have been conducted in Africa, a region with varying levels of urbanization between countries.

**Objective:**

To quantify the correlation between risk of child mortality and LAN as an indicator of urbanization and economic development in Africa.

**Methods:**

Using data from the Demographic and Health Survey (DHS) database conducted in 15 African countries out of 46 countries from 2005 to 2013, this study estimated LAN levels for children based on their year of birth and residential addresses. This study used Cox proportional hazards models to assess the association between LAN and the risk of child mortality in Africa.

**Results:**

The mean U5MR was 95 per 1,000 livebirths among the 15 African countries during 2005–2013. After adjusting for covariates, each 10-unit increment in LAN was associated with a 5.3% reduction in the risk of U5MR. The effect estimates were more pronounced in areas with lower LAN.

**Conclusion:**

In Africa, the risk of U5MR decreased with increasing LAN, especially in areas with lower LAN. The results suggest that the development of urbanization and socio-economic conditions may be beneficial to child health, especially in regions with low LAN. The use of LAN as a proxy may offer an intriguing approach for identifying areas requiring targeted development in urbanization and socio-economic conditions.

**Supplementary Information:**

The online version contains supplementary material available at 10.1186/s12889-023-17284-1.

## Introduction

The under 5 mortality rate (U5MR) is a widely used population health indicator, reflecting both the survival conditions of children and the broader social, economic, and environmental context in which they live. Both the Millennium Development Goals (MDGs) and the Sustainable Development Goals (SDGs) established by the United Nations (UN) have defined targets for decreasing U5MR. The SDGs specifically calls for the elimination of preventable newborn and child deaths [1], and all UN member states should work to reduce U5MR to 25 per 1,000 livebirths and below by 2030. Despite global progress in reducing child mortality over the past decades, disparities persist with many African countries continuing to experience high U5MR [2]. According to the report from United Nations International Children’s Emergency Fund, Sub-Saharan Africa had an average U5MR of 76 per 1,000 livebirths in 2019, nearly double the global average (38 per 1,000 livebirths), and approximately 15 times the average for Europe and Northern America (5 per 1,000 livebirths) [2]. Even if a series of public health and nutrition interventions have been taken, such as immunization and exclusive breastfeeding, high child mortality in Africa remains a big issue. Thus, more efforts are still needed to further reduce child mortality, especially in less developed regions.

In addition to traditional determinants of child mortality, such as malnutrition, infectious diseases, and injuries [3], socio-economic circumstances also influence child mortality risks, particularly in low-income countries [4]. Studies have demonstrated a robust link between poverty and elevated child mortality rates in Africa [5]. Moreover, child mortality is higher in rural areas compared to urban areas in low and middle-income countries [6, 7], indicating the potential beneficial impact of urbanization on children health [8].

Urbanization signifies a societal progression encompassing enhanced social productivity, scientific advancements, industrial restructuring, and the shift from rural to urban areas [9, 10]. This phenomenon involves changes in land use, infrastructure development, economic and social structures and demographic patterns. In previous studies, the spatiotemporal resolution of indicators to evaluate urbanization and economic development tend to be coarse, such as Gross Domestic Product (GDP), which is normally estimated on regional or national scale with low spatial resolutions. Some indicators of urbanization are binary, such as segregating regions as urban or rural in the Demographic and Health Survey (DHS). Such binary indicators are unable to capture the continuous change of socio-economic transformation from rural to urban in terms of regional economic development, increased population density, and infrastructure development [11–13]. Absolute wealth index derived from household goods in DHS could provide a more synthetic understanding of modernization level of a household [14]; however, these census-based urbanization indicators are often inaccessible in years and areas that are not surveyed. These limitations hinder uniform analysis on urbanization and economic development in developing regions at regional and continental levels, and underscore the need for easy-access indicators to comprehensively reflect the socio-economic conditions and urbanization dynamics in African region.

Light at night (LAN) from satellite retrieval products can reflect human lighting levels at night, and reveal human activity patterns and economic development at the earth surface. Satellite remote sensing LAN data are available for decades and can reflect the long-term trends and dynamics of urbanization and economic conditions in Sub-Saharan Africa [15]. Furthermore, LAN data has a higher spatial resolution of 1 km × 1 km, compared with traditional economic indicators like GDP. The satellite-based data can provide a uniform measuring method globally, facilitating multiple-location studies across country and continent. These advantages make LAN data an increasingly popular indicator for urbanization and socio-economic development. By measuring the intensity and spatiotemporal coverage of LAN, conducting comparative analyses across regions, and in conjunction with other socio-economic data, LAN has been used to determine the extent of urban areas in China [16], estimate the process of urbanization in India and globally [17, 18], and track population economic growth in Russia, Turkey and China [19–21]. Unlike traditional indicators of urban-rural dichotomy, LAN provides a more integrated picture of the extent of urban development, the magnitude of population density and the accessibility of transport.

While a growing number of researches have utilized LAN as an indicator of urbanization and socio-economic development, its association with the risk of child mortality has rarely been explored in Africa, a region with varying levels of urbanization between countries. This study aims to address these gaps by utilizing U5MR data from the African DHS survey and satellite LAN data to explore the association between LAN and the risk of U5MR in Africa. The findings of this study are expected to provide potential scientific evidence for protecting child health in the context of urbanization.

## Methods

### Study population

Data on child death in Africa is derived from Demographic and Health Survey (DHS) system, which provides nationally representative routine survey data from multiple countries in Africa. The DHS database is open access with approval at https://www.dhsprogram.com/ and has been used in previous studies [22–24]. This study included data from 15 countries through the combination of 32 datasets for analysis following the inclusive criteria: (1) surveys included information of women and child birth records, GPS data of their residential addresses and other covariates; (2) child birth year was during 2005–2013, as the DMSP-sourced LAN data was not available after 2013. Some countries were excluded from the analysis due to: (1) lack of coordinates system or Geographical dataset from their standard DHS; (2) the absence of significant variables available for comparison and analysis; (3) data from standard DHS collected before 2005. These 15 countries are located in Western Africa (Benin, Guinea, Mali, Nigeria), Central Africa (Cameroon, Chad), Eastern Africa (Burundi, Ethiopia, Tanzania, Uganda), and Southern Africa (Angola, Malawi, Zambia, Zimbabwe). Detailed information of the country selection process was presented in the supplementary material (Fig. S1).

### Definitions of health outcomes

The DHS questionnaire recorded the year of birth and duration of survival period at the time of the survey. Deaths under the age of five and infant deaths are defined as children who survived for less than 60 and 12 months, respectively [25].

### LAN data

LAN levels were estimated by retrievals from Linear Scanning Operational System (OLS) on board the United States DMSP nightlight satellite. DMSP was a program managed by the United States Defense that operated a series of satellites to collect meteorological, oceanographic and solar-terrestrial physics data. OLS onboard the DMSP satellites could be used for low-light imaging of the earth’s surface at night. The raw LAN data were processed by the National Geophysical Data Center (NGDC) of the United States Oceanic and Atmospheric Administration (NOAA) [26]. The processed data contains annual brightness of LAN from 1992 to 2013 at a spatial resolution of 1 km × 1 km and global coverage, which is available at https://eogdata.mines.edu/products/dmsp/. Due to the lack of on-board calibration mechanism, DMSP LAN product does not report values as radiance, but in Digital Number (DN) with values ranging from 0 to 63, indicating the intensity of LAN. The larger DN values indicate the brighter areas at night. The data are available to download in “tiff” format and in gridded cell forms with a resolution of 1 km × 1 km. The LAN values were matched with each child based on the annual average LAN in time corresponding to the year of birth, and in space corresponding to the grid cell that matched the latitude and longitude of the residential address. To perform sensible comparison between images of different years, intercalibration was performed according to the coefficient table provided by Earth Observation Group (EOG) at https://eogdata.mines.edu/products/dmsp/ [27]. Then, LAN was included in the model as a continuous variable.

### Covariates

The following factors mentioned in previous studies that may influence child mortality and be available in DHS were considered as covariates: maternal age, maternal smoking status, maternal education level, household cooking fuel, availability of toilet facilities, and accessibility to safe water. Maternal smoking status was categorized as yes/no, availability of toilet facilities and accessibility to safe water were categorized as improved/unimproved. The detailed definition of these parameters could be found in the DHS questionnaire, and we summarized them as described below. Maternal education level was categorized as low (no education/primary education) and high (secondary education and higher). Household cooking fuel was categorized as unclean (coal/lignite, charcoal, wood, straw/shrubs/grass, agriculture crops/animals dung, cardboard/paper) and clean (electricity, liquefied petroleum gas, natural gas and biogas, solar power). Availability of toilet facilities was categorized as improved (pit latrine with slab, VIP latrine, flush to septic tank, flush or pour flush toilet, flush to piped sewer system, covered pit latrine, bucket toilet, chemical toilets) and unimproved (bush, field). Accessibility to safe water was categorized as improved (protected well, protected spring, piped water, tanker truck, borehole with pump, bottled water, and public tap/standpipe) and unimproved (open wells, springs, and tube wells/boreholes). Household wealth index was a quantile of the household wealth (poorest/poorer/middle/richer/richest) extracted from DHS database, which is a composite measure of a household’s cumulative living standard calculated using easily collected data on a household’s cumulative ownership of selected assets.

### Statical analysis

The study used Cox proportional hazards model to explore the relationship between LAN and the risk of U5MR using child survival time as the outcome. We used a fully adjusted model to adjust for the following covariates that had been suggested by previous studies [4]: maternal smoking status, maternal education level, availability of toilet facilities, type of household cooking fuel, and accessibility to safe water, to estimate the association between LAN and U5MR more accurately. LAN was incorporated as a continuous variable in the model. The results were showed by hazard ratio (HR), representing the change in risk of U5MR associated with each 10-unit increase in LAN. Then the exposure-response curve between LAN and mortality risk was generated based on the Cox model.

To assess whether the association between LAN and the risk of child mortality varied by subpopulation, stratification analyses were conducted by child sex, maternal smoking status, maternal education level, household cooking fuel, toilet facilities and safe water accessibility. Then 95% CIs were applied to test the differences of effect estimates between two subgroups, *p* values for the potential effect modifiers were calculated from 95% CIs.

Several sensitivity analyses were carried out to verify the robustness of the results. Firstly, as the data version of LAN changed in 2010 with different satellite sensors, the main analyses were repeated using 2010 as the cut-off year to see if the effects were consistent in the datasets with child birth before 2010 (2005–2009) and after 2010 (2010–2013). Secondly, due to the skewed distribution of LAN, we used P60 (original DN = 0) as the cut-off and only included study subjects with LAN > P60 in the analysis. Thirdly, since infant death is also an important part of under-five deaths and a vital indicator of child health [2, 28], the analysis was repeated in subgroup of infant death.

All statistical analyses were performed using R software (version 3.6.3, Foundation for statistical Computing, Vienna, Austria), and cox regression models were conducted with the ‘Survival’ package.

## Results

### Descriptive results

During data processing, 0.24% of the study population was excluded due to the lack of geographic location data in the DHS dataset. Of 46 countries, 15 were included (Fig. S1), considering data accessibility and completeness of variables. Between 2005 and 2013, 557,930 children from 15 countries in Africa were finally included in the study. As shown in Table 1, the study population were mostly with mothers with low education level (77.46%), living in rural areas (71.51%), using unclean/biomass fuel for domestic cooking (92.85%), having improved toilet facilities (75.26%) and water accessibility (84.91%).

**Table 1:** Demographic characteristics and LAN exposure levels of the study population

Variable		N (%)	LAN (DN)	P value*
Maternal age, mean ± SD		31.77 ± 7.23	-	
Child sex				
	Male	271,703 (48.85)	7.51	
	Female	284,482 (51.15)	7.87	< 0.05
Maternal smoking status				
	No	493,182 (99.33)	7.64	
	Yes	3,350 (0.67)	11.34	0.05
Maternal education level				
	Low	432,130 (77.46)	5.06	
	High	125,780 (22.54)	16.62	< 0.05
Household cooking fuel				
	Unclean	510,634 (92.85)	5.89	
	Clean	39,341 (7.15)	30.41	< 0.05
Toilet facilities				
	Unimproved	136,067 (24.74)	2.53	
	Improved	413,960 (75.26)	9.32	< 0.05
Water				
	Unimproved	83,003 (15.09)	2.07	
	Improved	466,919 (84.91)	8.63	< 0.05
Access to health center				
	No	213,128 (42.38)	4.53	
	Yes	289,718 (57.62)	9.86	< 0.05
Living Area				
	Rural	398,985 (71.51)	2.29	
	Urban	158,945 (28.49)	21.16	< 0.05
Household wealth quantile				
	Poorest	130,466 (23.38)	2.07	
	Poorer	122,777 (22.01)	2.79	
	Medium	114,999 (20.61)	5.25	
	Richer	104,151 (18.67)	10.92	
	Richest	85,537 (15.33)	22.48	< 0.05

* *p* indicates the correlation between LAN and other demographic variables

Spatially, the overall LAN distribution was sparse and low in the study domain as shown in Table 2, Figs. S2 and S3. Of the 15 countries, the two countries with the highest overall brightness were South Africa and Nigeria, with mean LAN value of 1.48 and 1.12, respectively. The two countries with the lowest overall brightness were Chad and Mali, with mean LAN value of 0.01 and 0.03, respectively. In terms of temporal trend of LAN shown in Fig. S3, there was an overall upward trend in LAN in the 15 African countries from 2005 to 2013, with the mean value increasing from 0.45 in 2005 to 2.18 in 2013. Table 1 showed that LAN was higher in areas with children who had more educated mothers, clean fuels, improved toilet facilities and safe water, and lived in urban areas. Table 1 and Fig. S4 showed that LAN increased with higher household wealth quantile. The comparisons suggested that LAN was highly related to economic and urban development.

**Table 2:** Distribution of the study population based on sampling from DHS surveys during 2005–2013 and LAN level at country level in 15 African countries

Country	Total birth number	Death number of children under 5	Under 5 mortality rate (95%CI) (per 1,000 livebirths)	LAN* 2005	LAN 2013	Mean LAN (2005–2013)
Ethiopia	8,506	1,517	178 (170, 187)	0.08	1.91	0.77
Chad	34,023	4,385	129 (125, 132)	0.01	1.86	0.59
Nigeria	125,543	14,885	119 (117, 120)	1.12	2.87	1.99
Guinea	24,347	2,621	108 (104, 112)	0.06	1.91	0.75
Mali	35,614	3,541	99 (96, 103)	0.03	1.87	0.66
Cameroon	28,734	2,728	95 (92, 98)	0.11	1.95	0.84
Benin	38,698	3,336	86 (83, 89)	0.22	2.12	1.03
Uganda	56,467	4,653	82 (80, 85)	0.11	1.99	0.86
Burundi	30,471	2,365	78 (75, 81)	0.14	2.04	0.95
Malawi	52,671	3,966	75 (73, 78)	0.36	2.12	1.17
Zimbabwe	16,993	1,250	74 (70, 78)	0.33	2.07	1.08
South Africa	17,413	1,268	73 (69, 77)	1.48	3.22	2.43
Angola	21,323	1,527	72 (68, 75)	0.06	1.99	0.78
Zambia	42,643	3,027	71 (69, 73)	0.12	1.99	0.87
Tanzania	24,484	1,681	69 (66, 72)	0.09	1.93	0.80
Total	557,930	52,750	95 (94, 95)	0.45	2.18	1.29

*LAN refers to the average LAN of the corresponding country in DN units

A total of 52,750 under-five deaths occurred as shown in Table 2. The average U5MR within the study area was 95 per 1,000 livebirths, with the highest mortality rate of 178 per 1,000 livebirths in Ethiopia and the lowest rate of 69 per 1,000 livebirths in Tanzania. The average infant death rate was 66 per 1,000 livebirths during the study population.

### Regression results

**Fig. 1:**
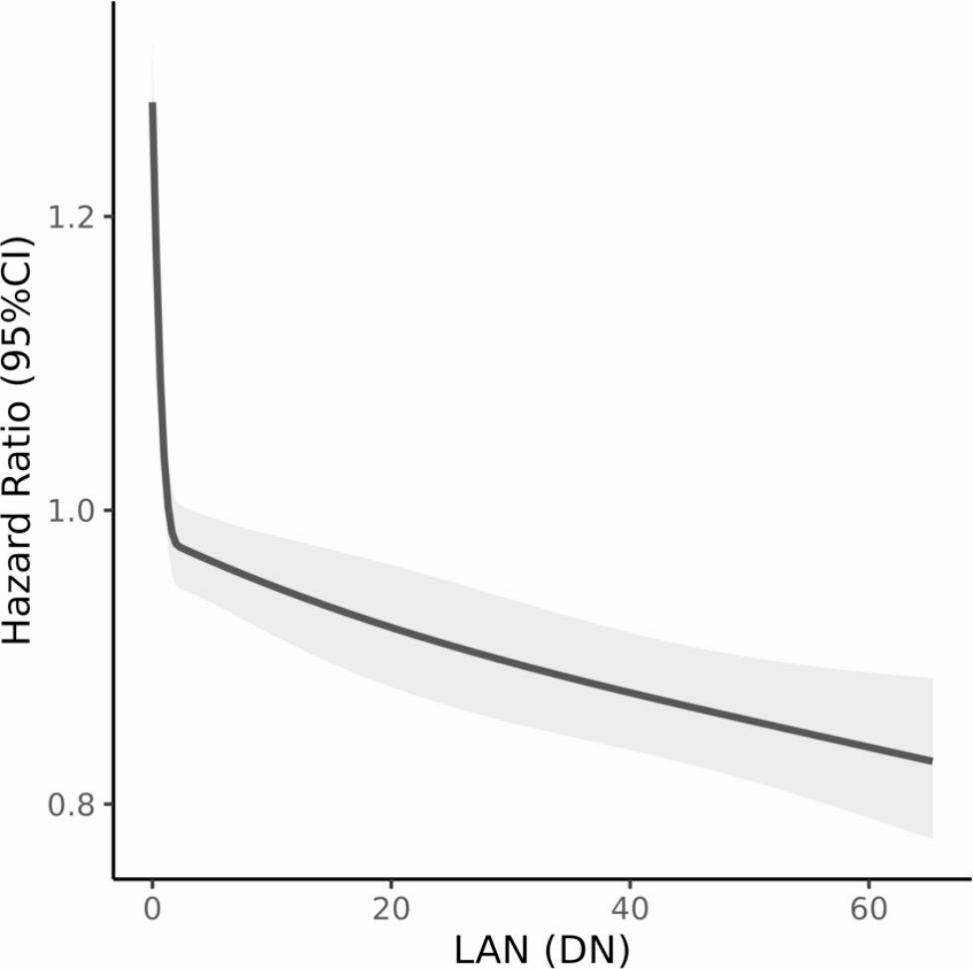
The exposure-response curves between risk of U5MR and LAN in 15 countries in Africa based on Cox regression analysis. Fully adjusted model adjusts for child sex, maternal age, maternal smoking status, maternal education, toilet facilities, fuel for domestic cooking, safe water accessibility

The risk of child mortality decreased with increasing LAN. For every 10-unit increment in LAN, the risk of U5MR decreased by 5.3% (HR: 0.947 with 95% CI [0.939, 0.954]). Fig. 1 displayed the exposure-response curve between HR and continuous LAN levels. The risk of child mortality decreased as LAN increased. When LAN < 2, the risk of child mortality decreased very rapidly along with the increase of LAN, showing a very steep slope of the curve; then, the risk of child mortality decreased more slowly and the curve flattened out.

In the stratification analyses, as shown in Table S2, there were no significant differences observed in effect estimates among subgroups based on maternal smoking status (yes/no), type of household cooking fuel (clean/unclean), and availability of toilet facilities (improved/unimproved). The effect estimates were significantly higher for children who were male, had less educated mothers and had unimproved water accessibility than for children who were female, had more educated mothers and improved water accessibility, respectively.

### Sensitivity analysis

After dividing dataset before 2010 and after 2010, the difference between effect estimates in 2005–2010 and in 2011–2013 was not statistically significant and the risk estimates were similar to that with data in 2005–2013. Result based on infant mortality also showed that the risk decreased by 3.2% for every 10-unit increase of LAN. Using data with LAN above P60, the effect estimates were similar to those with full dataset (Table 3). The HR value was 0.96 with data of LAN above P60 while 0.95 with full dataset.

**Table 3:** Sensitivity analysis of association between LAN and risk of child mortality in Africa

Variable	Child mortality	HR (95% CI) ^a^
Before 2010^b^	Under-five mortality	0.948 (0.938,0.958)
After 2010^c^	Under-five mortality	0.962 (0.949,0.975)
Infant death	Infant mortality	0.968 (0.958,0.977)
LAN (> P60)	Under-five mortality	0.963 (0.954,0.973)

^a^ HR values are expressed as HR per 10-unit increase in LAN; Models adjust for child sex, maternal age, maternal smoking status, maternal education, toilet facilities, fuel for domestic cooking, safe water accessibility

^b^ Before 2010 represents datasets with birth between 2005–2010;

^c^ After 2010 represents datasets with birth between 2011–2013

## Discussion

Of 557,930 children from 15 countries in Africa, lower LAN was associated with a higher risk of U5MR. The association was stronger in areas with lower LAN levels. Results were robust in sensitivity analysis.

Limited studies have examined the association between LAN and child mortality in Africa. LAN has several advantages over traditional economic and urbanization indicators like GDP and urban-rural dichotomy. With the long timescale (1992–2013) and the high spatial resolution (1 km × 1 km), LAN provides a more dynamic reflection of urbanization and socio-economic development, enabling precise exposure assessments. The consistent satellite-based LAN measurement approach enhances cross-country comparability, in contrast to the variability in criteria and definitions of traditional indicators. Previous research has successfully employed LAN as an indicator of urbanization and socio-economic development [18, 19, 29], demonstrating strong correlations with GDP, population density, economic status, and urban sprawl [15, 18, 21, 30, 31]. In our study, LAN was also found to be associated with socio-economic related variables, such as safe water accessibility, maternal education, and household wealth quartiles. Therefore, we employed LAN as a proxy for socio-economic conditions and urbanization. However, the application of LAN also has its limitations. For instance, DMSP measures the intensity of LAN, but not its spectral composition or source. It is also possible that some areas with high-intensity LAN levels not related to human activity, but rather to natural sources of LAN, such as wildfires or volcanic eruptions. Temporally, LAN data from DMSP is only updated to 2013, despite the availability of updated LAN products, the units for the two are different and the data cannot be well connected.

Per 10-unit increase in LAN was found to be associated with a 5.3% reduction in U5MR and a 3.2% reduction in infant mortality in Sub-Saharan Africa, suggesting that urbanization and socio-economic development may be beneficial to child health. These findings are consistent with previous studies using other indicators of economic development and urbanization. Many studies had also found the significant impact of socio-economic development on the risk of child mortality. In an analysis of DHS data, the level of economic development was strongly associated with child health, and the effect was greater in low-income countries [32]. For example, a study found every 10% increase in GDP was associated with a 6% reduction in all-cause mortality among infants [6].

The results showed that higher LAN level was associated with lower U5MR, and the association was stronger in areas with extremely low LAN levels in Africa. As shown in the exposure-response curve fitted to LAN and child mortality risk, the correlation between elevated LAN and reduced mortality was stronger in low LAN areas. This may be related to the finding that slums in large cities may be deleterious to child health in previous studies [33, 34]. The resolution of LAN was relatively coarse and could not distinguish urban slums. Therefore, it is difficult to quantify whether the LAN elevation within metropolitan would produce negative effects on child health. The results of our study should be interpreted with caution, and further studies are needed to explore the non-linear relationship. In the stratified analysis, for children with lower maternal education and unimproved water accessibility, changes in LAN for the same level were associated with a higher change in decreased risk of death, which also supported that those subgroups with poorer LAN exposure may be more susceptible and gain greater health benefits from the improvement of urbanization and socio-economic development in the future. As a study showed, every 1% increase in per capita health expenditure in sub-Saharan Africa was associated with a 0.5% reduction for U5MR [35]. However, the World Health Organization reported that health expenditures were generally low and only accounted for a small fraction of GDP in sub-Saharan Africa compared to those invested in developed economies. For example, in 2010, health expenditures in sub-Saharan Africa were only 6% of GDP, while such expenditures were 13-17% of GDP in Organization for Economic Co-operation and Development (OCED) countries and North America [35, 36]. Thus, there is a potential for greater health benefits through strategic investments in healthcare in regions with lower LAN levels.

The study has several limitations. Firstly, there may be exposure misclassification for the study population. LANs were matched based on the children’s birth time and the current address in their mother’s records in the DHS survey, but the change of address after birth was not recorded in the DHS database. Secondly, we used LAN as an indicator of urbanization and economic development in this study. Although LAN is a continuous variable with long temporal coverage and high spatial resolution, it could not provide accurate estimates of social economic status (SES) at individual level. We have conducted multiple sensitive analyses to validate our results, but studies with high-quality SES parameters at individual level are needed to further validate our findings. Thirdly, the relationship between child health and LAN was also complex, the information on the specific cause of child death was not collected, and infectious diseases such as HIV/AIDS may also have an impact on this relationship, which needed to be further explored. Moreover, certain factors such as handwashing, which may contribute to respiratory and intestinal infectious diseases, were not included in our study and merit further discussion in this context. Fourthly, DHS surveys rely on self-reporting by the household members, and in some cases, respondents may over-report or under-report the answer. Sampling bias may also occur in the survey process.

## Conclusion

The risk of U5MR was negatively associated with LAN levels, and the association was much stronger in lower LAN areas. Our results suggest that urbanization may have a positive impact on child health outcomes in certain contexts. These findings extend the application of LAN as a proxy for socio-economic indicators to explore its relationship with the risk of child mortality.

### Electronic supplementary material

Below is the link to the electronic supplementary material.

**Supplementary Material 1: Figure S1.** Flow diagram of the country selection progress in the analysis. **Figure S2.** Distribution of LAN in Africa in 2005. **Figure S3.** Changes in annual mean LAN in the 15 countries included in this study from 2005 to 2013. **Figure S4.** Correlation between household wealth quantile and LAN in the 15 countries included in this study. **Table S1.** Distribution of Under 5 mortality rate and LAN level for the study population in urban and rural areas in 15 African countries. **Table S2.** Stratified analysis of LAN and risk of child mortality in Africa

## Data Availability

All data were obtained from the public open databases: the Demographic and Health Survey (DHS) database (https://www.dhsprogram.com/) and DMSP-OLS Nighttime Lights Database (https://eogdata.mines.edu/products/dmsp/).

## References

[1] Hug L (2019). National, regional, and global levels and trends in neonatal mortality between 1990 and 2017, with scenario-based projections to 2030: a systematic analysis. The Lancet Global Health.

[2] WHO. UN-IGME-child-mortality-report-2020.pdf. 2021; Available from: https://www.unicef.org.

[3] Black RE, Morris SS, Bryce J (2003). Where and why are 10 million children dying every year?. The Lancet.

[4] Schell CO (2007). Socioeconomic determinants of infant mortality: a worldwide study of 152 low-, middle-, and high-income countries. Scand J Public Health.

[5] Worku EB, Woldesenbet SA (2015). Poverty and inequality - but of what - as social determinants of health in Africa?. Afr Health Sci.

[6] Ward JL, Viner RM (2017). The impact of income inequality and national wealth on child and adolescent mortality in low and middle-income countries. BMC Public Health.

[7] Van de Poel E, O’Donnell O, Van Doorslaer E. Are urban children really healthier? Evidence from 47 developing countries. Social Science & Medicine; 2007;65(10):1986–2003. 10.1016/j.socscimed.2007.06.032 17698272

[8] Antai D, Moradi T (2010). Urban area disadvantage and Under-5 mortality in Nigeria: the Effect of Rapid Urbanization. Environ Health Perspect.

[9] Christiaensen L, Todo Y (2014). Poverty reduction during the Rural-Urban Transformation - the role of the Missing Middle. World Dev.

[10] Zhang X et al. Linking urbanization and air quality together: a review and a perspective on the future sustainable urban development. J Clean Prod, 2022. 346.

[11] Calì M, Menon C (2013). Does Urbanization Affect Rural Poverty? Evidence from Indian districts. World Bank Econ Rev.

[12] Cohen B (2004). Urban growth in developing countries: a review of current trends and a caution regarding existing forecasts. World Dev.

[13] McDade TW, Adair LS. Defining the urban in urbanization and health: a factor analysis approach. Social Sci & Med. 2001;53(1):55–70. 10.1016/s0277-9536(00)00313-0 11380161

[14] Hohmann S, Garenne M. Absolute versus relative measures of poverty. Application to DHS African surveys. J US-China Public Administration 2011:8.

[15] Storeygard A (2016). Farther on down the road: transport costs, trade and urban growth in sub-saharan Africa. Rev Econ Stud.

[16] Ma T (2015). Night-time light derived estimation of spatio-temporal characteristics of urbanization dynamics using DMSP/OLS satellite data. Remote Sens Environ.

[17] Small C, Pozzi F, Elvidge C (2005). Spatial analysis of global urban extent from DMSP-OLS night lights. Remote Sens Environ.

[18] Pandey B, Joshi PK, Seto KC (2013). Monitoring urbanization dynamics in India using DMSP/OLS night time lights and SPOT-VGT data. Int J Appl Earth Obs Geoinf.

[19] Bennett MM, Smith LC (2017). Advances in using multitemporal night-time lights satellite imagery to detect, estimate, and monitor socioeconomic dynamics. Remote Sens Environ.

[20] Ortakavak Z (2020). Determination of the nighttime light imagery for urban city population using DMSP-OLS methods in Istanbul. Environ Monit Assess.

[21] Li XM, Zhou WQ (2018). Dasymetric mapping of urban population in China based on radiance corrected DMSP-OLS nighttime light and land cover data. Sci Total Environ.

[22] Abay KA, Amare M (2018). Night light intensity and women’s body weight: evidence from Nigeria. Econ Hum Biol.

[23] Amare M, et al. Urbanization and child nutritional outcomes. The World Bank Economic Review; 2018.

[24] Filmer D, Pritchett L (1999). The effect of household wealth on educational attainment: evidence from 35 countries. Popul Dev Rev.

[25] CDC. Infant Mortality. 2018; Available from: https://www.cdc.gov/reproductivehealth/MaternalInfantHealth/InfantMortality.htm/.

[26] Baugh K et al. *Development of a 2009 Stable Lights Product using DMSP-OLS data* Proceedings of the Asia-Pacific Advanced Network, 2010. 30(0).

[27] *The coefficient table for intercalibration*. 2021; Available from: https://eogdata.mines.edu/dmsp/v4c_2nd_order_sicily_x1to62_y1to63.20160114.csv.

[28] You D (2010). Levels and trends in child mortality, 1990–2009. Lancet.

[29] *Modeling population density with night-time satellite imagery and GIS*

[30] Li X (2017). Intercalibration between DMSP/OLS and VIIRS night-time light images to evaluate city light dynamics of Syria’s major human settlement during Syrian civil War. Int J Remote Sens.

[31] Li X (2015). Detecting 2014 northern Iraq Insurgency using night-time light imagery. Int J Remote Sens.

[32] Boyle MH (2006). The influence of economic development level, household wealth and maternal education on child health in the developing world. Soc Sci Med.

[33] Garenne M (2010). Urbanisation and child health in resource poor settings with special reference to under-five mortality in Africa. Arch Dis Child.

[34] Kimani-Murage EW (2014). Trends in childhood mortality in Kenya: the urban advantage has seemingly been wiped out. Health Place.

[35] Nketiah-Amponsah E (2019). The impact of Health expenditures on Health outcomes in Sub-saharan Africa. J Developing Soc.

[36] Mathee A (2011). Environment and health in South Africa: gains, losses, and opportunities. J Public Health Policy.

