# Supplementary material for: Association between light at night and the risk of child death in sub-saharan Africa: a cross-sectional analysis based on DHS data
Source: BMC Public Health. 2023 Nov 29;23:2366. doi: 10.1186/s12889-023-17284-1 (PMC10685554; doi:10.1186/s12889-023-17284-1)
Supplement: Supplementary file 1 — Supplementary Material 1: Figure S1. Flow diagram of the country selection progress in the analysis. Figure S2. Distribution of LAN in Africa in 2005. Figure S3. Changes in annual mean LAN in the 15 countries included in this study from 2005 to 2013. Figure S4. Correlation between household wealth quantile and LAN in the 15 countries included in this study. Table S1. Distribution of Under 5 mortality rate and LAN level for the study population in urban and rural areas in 15 African countries. Table S2. Stratified analysis of LAN and risk of child mortality in Africa [file 12889_2023_17284_MOESM1_ESM.docx]

**Supplementary Material**

**Association between light at night and the risk of child death in Sub-Saharan Africa: a cross-sectional analysis based on DHS data**

Xinyue Li^a^, Jovine Bachwenkizi^b^, Renjie Chen^a^, Haidong Kan^a^, Xia Meng^a*^

**Author Affiliations:**

1. School of Public Health, Key Laboratory of Public Health Safety of the Ministry of Education and Key Laboratory of Health Technology Assessment of the Ministry of Health, Fudan University, Shanghai 200302, China.
2. Department of Environmental and Occupational Health, Muhimbili University of Health and Allied Sciences, Dar es Salaam, Tanzania.

***Corresponding authors:**

Xia Meng, School of Public Health, Key Laboratory of Public Health Safety of the Ministry of Education and Key Laboratory of Health Technology Assessment of the Ministry of Health, Fudan University, Shanghai 200032, China. Email: mengxia@fudan.edu.cn;

**Table of content**

Figure S1*.* Flow diagram of the country selection progress in the analysis.

Figure S2. Distribution of LAN in Africa in 2005.

Figure S3. Changes in annual mean LAN in the 15 countries included in this study from 2005 to 2013.

Figure S4. Correlation between household wealth quantile and LAN in the 15 countries included in this study.

Table S1. Distribution of Under 5 mortality rate and LAN level for the study population in urban and rural areas in 15 African countries.

Table S2. Stratified analysis of LAN and risk of child mortality in Africa.

**
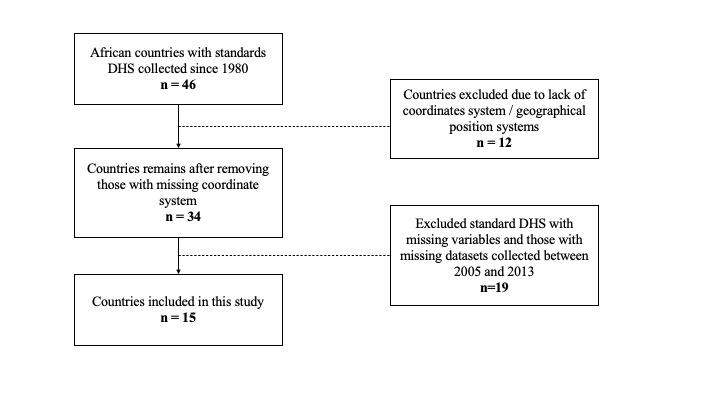
**

Figure S1*.* Flow diagram of the country selection progress in the analysis.


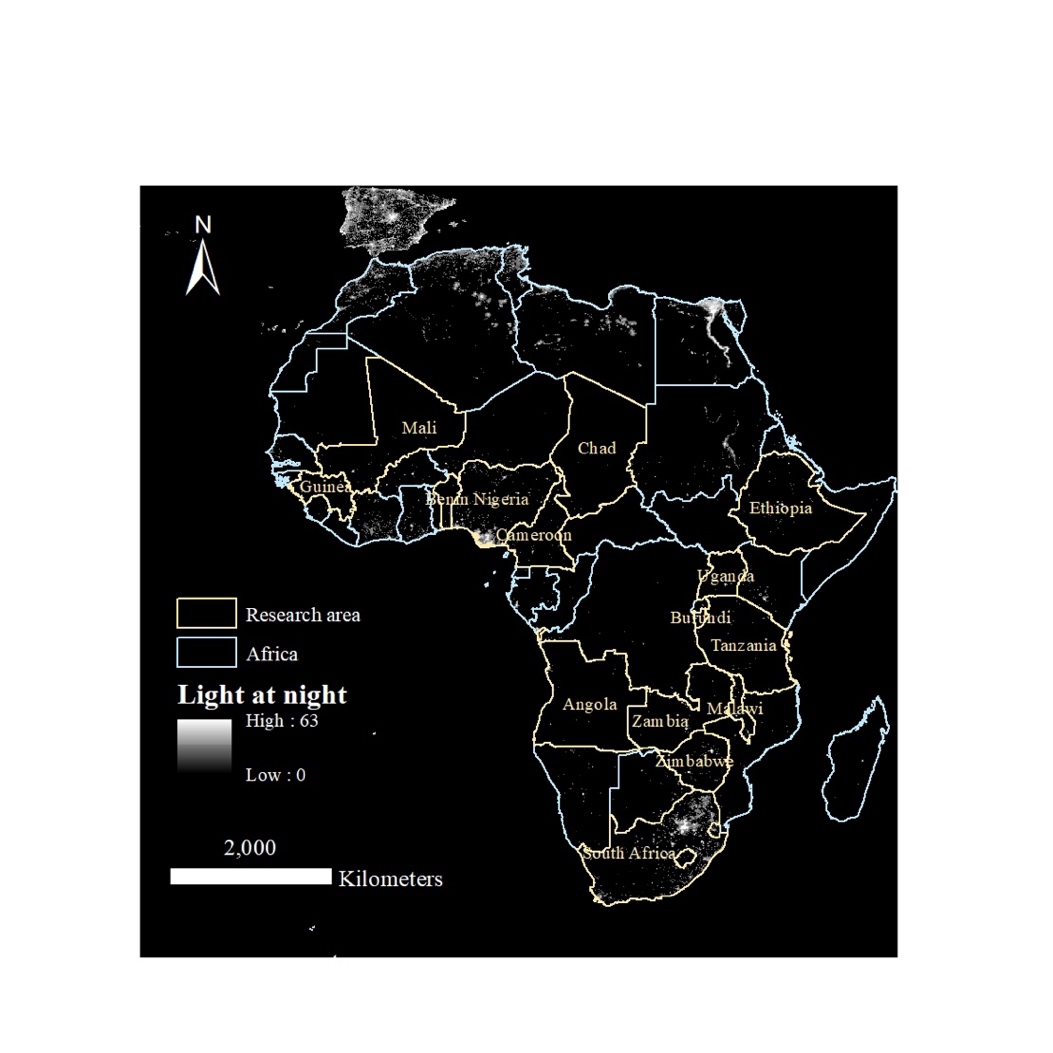


Figure S2. Distribution of LAN in Africa in 2005.


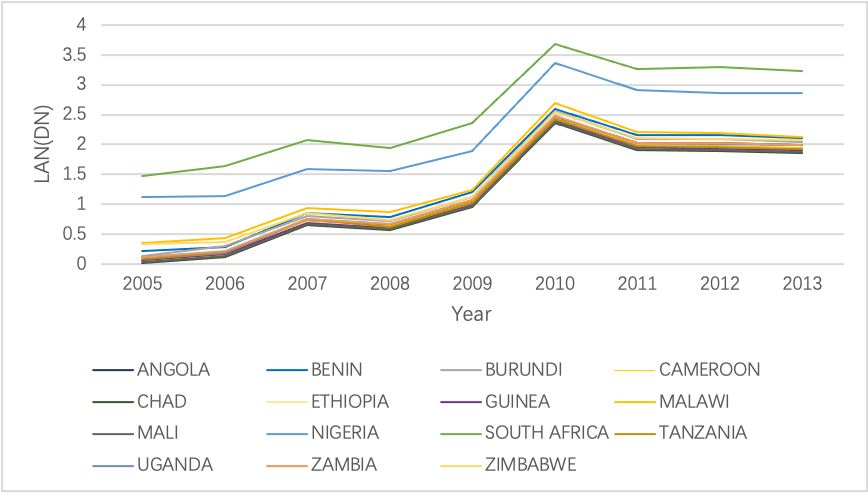


Figure S3. Changes in annual mean LAN in the 15 countries included in this study from 2005 to 2013.


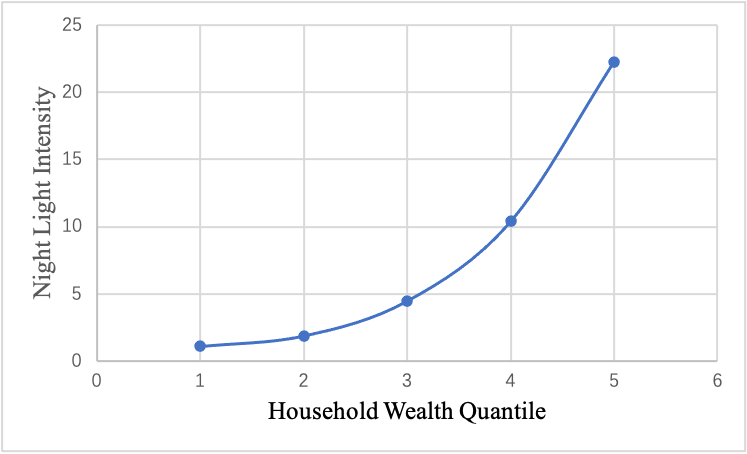


Figure S4. Correlation between household wealth quantile and LAN in the 15 countries included in this study. Household wealth index was estimated from household’s cumulative ownership of selected assets in the Demographic and Health Survey.

Table S1. Distribution of Under 5 mortality rate and LAN level for the study population in urban and rural areas in 15 African countries.

| Country | Urban LAN# | Rural LAN# | Urban under 5 mortality rate (95% CI) (1/1,000) | Rural under 5 mortality rate (95% CI) (1/1,000) |
| --- | --- | --- | --- | --- |
| Angola | 29.01 | 1.86 | 64 (59, 68) | 82 (76, 87) |
| Benin | 17.27 | 2.21 | 73 (68, 77) | 94 (91, 98) |
| Burundi | 11.14 | 1.16 | 59 (52, 66) | 81 (78, 85) |
| Cameroon | 20.07 | 1.28 | 75 (70, 80) | 109 (105, 114) |
| Chad | 13.05 | 1.18 | 127 (120, 135) | 129 (125, 133) |
| Ethiopia | 21.96 | 0.64 | 123 (107, 140) | 191 (182, 200) |
| Guinea | 15.82 | 1.28 | 73 (67, 80) | 120 (116, 125) |
| Malawi | 19.24 | 1.33 | 62 (57, 68) | 77 (75, 80) |
| Mali | 32.72 | 1.66 | 62 (57, 67) | 112 (108, 115) |
| Nigeria | 20.24 | 2.88 | 82 (80, 85) | 136 (133, 138) |
| South Africa | 39.68 | 9.55 | 66 (61, 71) | 80 (74, 86) |
| Tanzania | 15.02 | 1.60 | 77 (70, 85) | 66 (63, 70) |
| Uganda | 10.36 | 4.31 | 66 (62, 71) | 86 (83, 88) |
| Zambia | 22.79 | 1.76 | 72 (68, 77) | 70 (67, 73) |
| Zimbabwe | 24.50 | 1.77 | 66 (60, 73) | 77 (72, 82) |
| Total | 21.16 | 2.29 | 75 (74, 76) | 102 (101, 103) |

^#^: Urban refers to the study population in urban area for each country; Rural refers to the study population in rural area for each country.

Table S2. Stratified analysis of LAN and risk of child mortality in Africa.

| Variable | Group | HR（95%CI） |
| --- | --- | --- |
| Child sex ^*^ | |  |
|  | Male | 0.935 (0.924,0.945) |
|  | Female | 0.961 (0.973,0.949) |
| Mother's smoking status | |  |
|  | Yes | 0.895 (0.821, 0.977) |
|  | No | 0.947 (0.939, 0.955) |
| Mother's education level ^*^ | |  |
|  | Low | 0.934 (0.925, 0.944) |
|  | High | 0.971 (0.958, 0.985) |
| Household cooking fuel | |  |
|  | Unclean | 0.957 (0.937, 0.978) |
|  | Clean | 0.945 (0.936, 0.953) |
| Toilet facilities | |  |
|  | Unimproved | 0.947 (0.939, 0.955) |
|  | Improved | 0.949 (0.920, 0.979) |
| Safe water ^*^ | |  |
|  | Unimproved | 0.949 (0.941, 0.957) |
|  | Improved | 0.883 (0.835, 0.932) |
| Living area |  |  |
|  | Urban | 0.967 (0.957, 0.978) |
|  | Rural | 0.953 (0.934, 0.973) |

^a^ HR values are expressed as HR per 10-unit increase in LAN;

^*^ indicates p-values < 0.05, P values for the potential effect modifiers were calculated from 95% CIs.
